# Hyperbilirubinemia as an Indicator of Complicated Appendicitis

**DOI:** 10.7759/cureus.114685

**Published:** 2026-08-17

**Authors:** Abdelrahman Ibrahim, Raneem El-Jabri, Laura Awad, Abdrabbou N Mashhour, George Abdelfady Nashed, Ahmed M Hassan

**Affiliations:** 1 Surgery, Cairo University Hospitals, Cairo, EGY; 2 Surgery, Health Education North West, Preston, GBR; 3 Surgery, Health Education England, Manchester, GBR

**Keywords:** acute complicated appendicitis, appendicitis, complicated appendicitis, hyperbilirubinemia, unconjugated bilirubin

## Abstract

Background: Reliable preoperative identification of complicated appendicitis may support appropriate selection for operative or non-operative pathways. This study evaluated admission total serum bilirubin as an adjunctive marker of complicated disease and compared its performance with white blood cell count (WBC).

Methods: We prospectively enrolled 150 patients who underwent appendicectomy for suspected acute appendicitis at Cairo University Hospital over six months. Operative findings classified cases as negative appendicectomy, uncomplicated appendicitis, or complicated appendicitis. Diagnostic accuracy was assessed among patients with operative appendicitis. Receiver operating characteristic (ROC) analysis was used to select thresholds based on the maximum Youden index. Exact Clopper-Pearson 95% confidence intervals (CIs) were calculated for sensitivity, specificity, positive predictive value (PPV), and negative predictive value (NPV).

Results: Of 150 participants, 35 (23.3%) had a macroscopically normal appendix, 86 (57.3%) had uncomplicated appendicitis, and 29 (19.3%) had complicated appendicitis. For complicated versus uncomplicated appendicitis, total bilirubin had an area under the ROC curve (AUC) of 0.904 (bootstrap 95% CI 0.827-0.968; P < 0.001). At a threshold of ≥0.95 mg/dL, sensitivity was 82.76% (95% CI 64.23%-94.15%), specificity 94.19% (86.95%-98.09%), PPV 82.76% (64.23%-94.15%), and NPV 94.19% (86.95%-98.09%). WBC had an AUC of 0.717 (95% CI 0.596-0.826; P < 0.001); at ≥14.3 x 10⁹/L, sensitivity was 72.41% and specificity 76.74%.

Conclusion: Admission total serum bilirubin showed good discrimination for complicated appendicitis in this single-center operative cohort and performed better than WBC alone. It should be interpreted as an adjunct to clinical assessment and imaging, and the identified threshold requires independent validation.

## Introduction

Acute appendicitis remains one of the most frequent causes of emergency abdominal surgery. Diagnosis is usually based on the combined interpretation of symptoms, examination findings, laboratory markers, clinical scores, and imaging; nevertheless, atypical anatomy and overlapping gastrointestinal or genitourinary presentations can make the diagnosis uncertain [1,2].

The distinction between uncomplicated and complicated appendicitis has gained particular importance as antibiotic-first treatment may be considered for selected patients with uncomplicated disease [3,4]. Complicated appendicitis generally includes gangrene, perforation, periappendiceal abscess, or peritonitis and commonly requires more urgent source control and broader perioperative management [5]. Preoperative classification remains imperfect because inflammatory markers are non-specific and imaging features of perforation are not always present [6-9].

Hyperbilirubinemia has been proposed as a marker of severe appendiceal inflammation. Bacterial translocation, endotoxin exposure, and cytokine-mediated impairment of hepatocellular transport may contribute to transient cholestasis during advanced intra-abdominal infection [10,11]. Previous studies have reported variable sensitivity but generally higher specificity for bilirubin than for leukocytosis when predicting perforation or gangrene [10-13].

The primary objective of this study was to determine the diagnostic accuracy of admission total serum bilirubin for identifying complicated appendicitis among patients with operatively confirmed appendicitis. A secondary objective was to compare its performance with white blood cell count (WBC) and to explore the ability of both markers to distinguish uncomplicated appendicitis from negative appendicectomy.

## Materials and methods

Study design and setting

This prospective, single-center diagnostic-accuracy study was conducted over a six-month period at Cairo University Hospital, a tertiary referral center for emergency general surgery. Patients undergoing appendicectomy for a clinical diagnosis of acute appendicitis were considered for enrolment. Data were recorded at admission by one investigator and independently checked by a second investigator before analysis.

Ethical approval

Ethical approval was obtained from the Faculty of Medicine, Kasr Al Ainy, Cairo University, for the study protocol entitled “Correlation between acute appendicitis and level of serum bilirubin” on October 17, 2017. No study-specific ethics approval reference number was issued at that time. Written informed consent was obtained from all participants before enrolment in the study.

Eligibility and clinical assessment

Patients were eligible when they underwent open or laparoscopic appendicectomy for suspected acute appendicitis. Patients with known hepatobiliary disease, hemolytic anemia, or congenital hyperbilirubinemia were excluded because these conditions could independently increase serum bilirubin levels. Patients with an appendiceal mass managed conservatively were not included unless subsequent surgery was required following failure of non-operative treatment.

All participants underwent clinical assessment and Alvarado scoring [14]. Admission investigations included a complete blood count, total and direct serum bilirubin, alanine aminotransferase, aspartate aminotransferase (AST), urea, creatinine, electrolytes, and coagulation tests. Blood samples were analyzed at the Kasr Al Ainy Hospital New Emergency Laboratory. The laboratory reference interval for WBC was 4-11 × 10⁹/L. The biochemical reference intervals were total serum bilirubin up to 0.9500 mg/dL, AST up to 37 U/L, blood urea 15-45 mg/dL, serum creatinine 0.40-1.30 mg/dL, and serum sodium 132-148 mmol/L. The thresholds identified by the receiver operating characteristic (ROC) analyses were data-derived exploratory diagnostic cut-offs and did not represent the laboratory reference limits.

Abdominopelvic ultrasonography was used as an adjunct according to local practice but was not used as the reference standard. The choice of open or laparoscopic appendicectomy followed local practice informed by Society of American Gastrointestinal and Endoscopic Surgeons (SAGES) guidance [15].

Reference standard and outcome classification

The reference standard was the surgeon’s macroscopic intraoperative assessment. A macroscopically normal appendix was classified as a negative appendicectomy. Uncomplicated appendicitis was defined by inflammation, hyperemia, and edema without necrosis, perforation, abscess, or peritonitis. Complicated appendicitis was defined by one or more of the following findings: gangrene or tissue necrosis, perforation, periappendiceal abscess, or peritonitis, consistent with published operative definitions [5]. Routine histopathological confirmation was not available at the study center.

Statistical analysis

Statistical analyses were performed using IBM SPSS Statistics for Windows, Version 25.0 (IBM Corp., Armonk, NY, USA) and were independently reproduced from the study dataset. Continuous variables were summarized using means and standard deviations or medians and ranges, as appropriate. Categorical variables were summarized as counts and percentages. Kruskal-Wallis and Mann-Whitney U tests were used for non-normally distributed continuous variables [16]. Standard diagnostic indices, including sensitivity, specificity, predictive values, likelihood ratios, and accuracy, were calculated from 2 × 2 contingency tables [17].

ROC curves were constructed for total serum bilirubin and WBC. The optimal cut-off was defined as the value that maximized the Youden index (sensitivity + specificity − 1). The 95% confidence intervals (CIs) for the area under the ROC curve (AUC) were estimated using non-parametric bootstrap resampling with 5,000 repetitions. P-values assessed whether marker distributions differed between outcome groups using the two-sided Mann-Whitney U test, which is equivalent to testing whether the AUC differs from 0.50. Sensitivity, specificity, positive predictive value (PPV), and negative predictive value (NPV) were calculated from 2 × 2 contingency tables. Exact Clopper-Pearson binomial 95% CIs were applied consistently to all four proportions, while likelihood-ratio CIs were estimated on the logarithmic scale. Statistical significance was set at P < 0.05.

## Results

Participant characteristics

The cohort included 150 participants: 75 men and 75 women. Mean age was 27.8 years (range 12-72 years; median 25 years). Operative assessment identified 35 negative appendicectomies (23.3%), 86 cases of uncomplicated appendicitis (57.3%), and 29 cases of complicated appendicitis (19.3%) (Table 1).

**Table 1:** Demographic and operative classification of the study cohort.

Characteristic	Category	n	%
Sex	Male	75	50.0
	Female	75	50.0
Operative diagnosis	Negative appendicectomy	35	23.3
	Uncomplicated appendicitis	86	57.3
	Complicated appendicitis	29	19.3
	Total	150	100.0

Diagnostic performance of WBC

Among the 115 patients with operative appendicitis, a WBC threshold of ≥14.3 x 10⁹/L identified 21 of 29 complicated cases and was positive in 20 of 86 uncomplicated cases (Table 2). Sensitivity was 72.41% (95% CI 52.76%-87.27%), specificity 76.74% (66.39%-85.18%), PPV 51.22% (35.13%-67.12%), NPV 89.19% (79.80%-95.22%), and accuracy 75.65% (66.77%-83.17%) (Table 3).

**Table 2:** Cross-classification of WBC at the ROC-derived threshold. WBC: white blood cell count; ROC: receiver operating characteristic

WBC	Complicated, n (%)	Uncomplicated, n (%)
≥14.3 x 10⁹/L	21 (72.4)	20 (23.3)
<14.3 x 10⁹/L	8 (27.6)	66 (76.7)
Total	29 (100.0)	86 (100.0)

**Table 3:** Diagnostic accuracy of WBC for complicated appendicitis. WBC: white blood cell count; CI: confidence interval; AUC: area under the ROC curve; ROC: receiver operating characteristic

Measure	Estimate	95% CI
Sensitivity	72.41%	52.76-87.27
Specificity	76.74%	66.39-85.18
Positive likelihood ratio	3.11	2.00-4.86
Negative likelihood ratio	0.36	0.20-0.66
Positive predictive value	51.22%	35.13-67.12
Negative predictive value	89.19%	79.80-95.22
Accuracy	75.65%	66.77-83.17
AUC	0.717	0.596-0.826

Diagnostic performance of total bilirubin

At a threshold of ≥0.95 mg/dL, total bilirubin was positive in 24 of 29 patients with complicated appendicitis and in five of 86 patients with uncomplicated appendicitis (Table 4). Sensitivity and PPV were both 82.76% (exact 95% CI 64.23%-94.15%), while specificity and NPV were both 94.19% (86.95%-98.09%). The identical point estimates have identical exact intervals because the relevant numerators and denominators are also identical in this dataset. Overall accuracy was 91.30% (84.59%-95.75%).

**Table 4:** Diagnostic accuracy of total serum bilirubin for complicated appendicitis. CI: confidence interval; AUC: area under the ROC curve; ROC: receiver operating characteristic

Measure	Estimate	95% CI
Sensitivity	82.76%	64.23-94.15
Specificity	94.19%	86.95-98.09
Positive likelihood ratio	14.23	5.98-33.87
Negative likelihood ratio	0.18	0.08-0.41
Positive predictive value	82.76%	64.23-94.15
Negative predictive value	94.19%	86.95-98.09
Accuracy	91.30%	84.59-95.75
AUC	0.904	0.827-0.968

In Table 5, the cut-off value for total serum bilirubin levels for diagnosing complicated appendicitis was 0.95 mg/dL. At the ROC-derived threshold of ≥0.95 mg/dL, total bilirubin was elevated in 24 of 29 patients with complicated appendicitis and in five of 86 patients with uncomplicated appendicitis. This corresponded to a sensitivity of 82.8% and specificity of 94.2%.

**Table 5:** Cross-classification of total bilirubin at the ROC-derived threshold. ROC: receiver operating characteristic

Total bilirubin	Complicated, n (%)	Uncomplicated, n (%)
≥0.95 mg/dL	24 (82.8)	5 (5.8)
<0.95 mg/dL	5 (17.2)	81 (94.2)
Total	29 (100.0)	86 (100.0)

ROC curve for WBCs and total serum bilirubin

**Figure 1:**
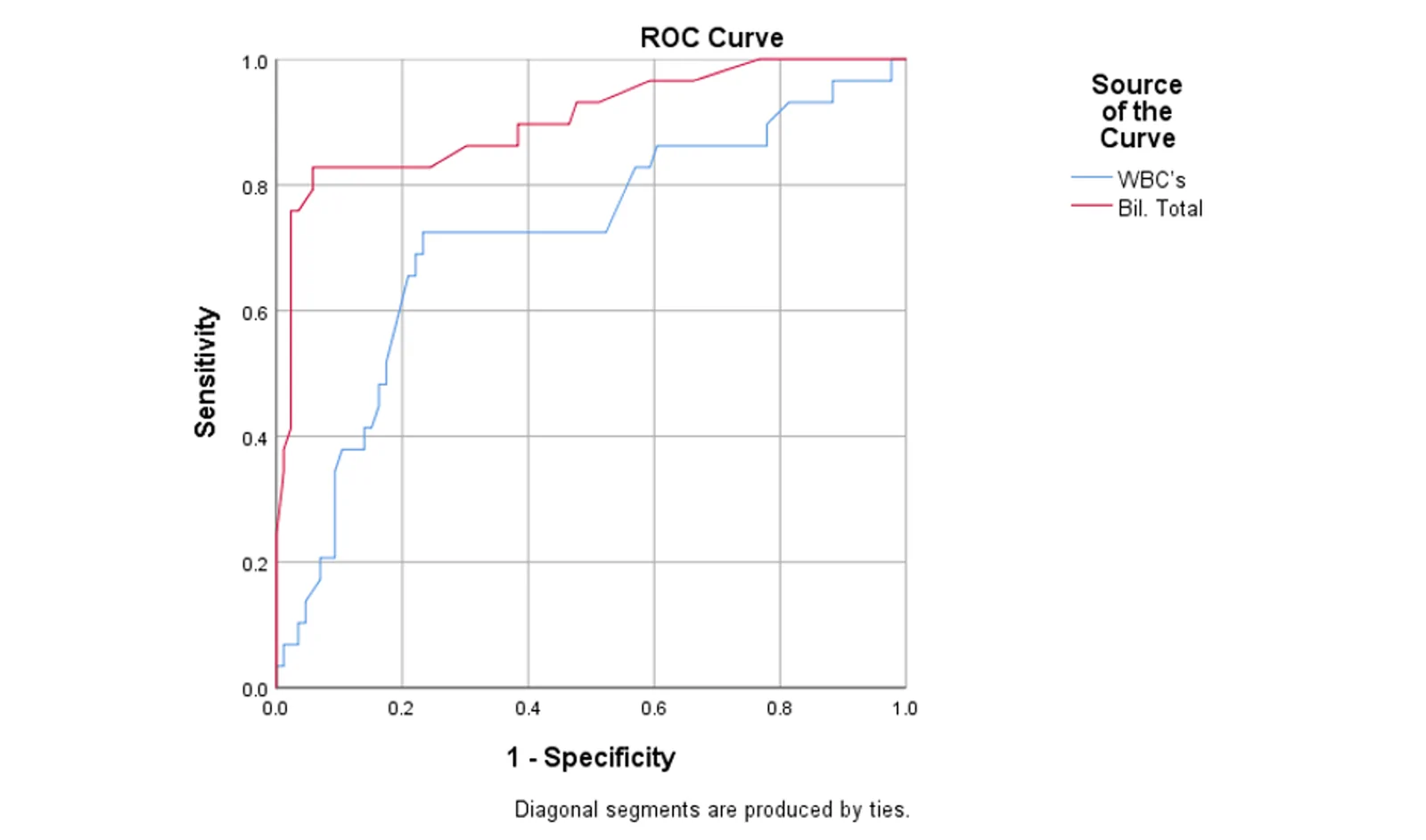
ROC curves for WBC and total serum bilirubin in distinguishing complicated from uncomplicated appendicitis. Bilirubin AUC 0.904 (95% CI 0.827-0.968; P < 0.001); WBC AUC 0.717 (95% CI 0.596-0.826; P < 0.001). Optimal cut-offs were selected by the maximum Youden index. CI: confidence interval; AUC: area under the ROC curve; ROC: receiver operating characteristic; WBC: white blood cell count

For distinguishing complicated from uncomplicated appendicitis, total bilirubin produced an AUC of 0.904 (bootstrap 95% CI 0.827-0.968; P < 0.001), compared with 0.717 for WBC (0.596-0.826; P < 0.001). The maximum Youden index supported thresholds of ≥0.95 mg/dL for bilirubin and ≥14.3 x 10⁹/L for WBC. The corresponding curves are presented in Figure 1.

## Discussion

This prospective study found that admission total serum bilirubin was strongly associated with complicated appendicitis among patients undergoing appendicectomy. At the ROC-derived threshold, bilirubin achieved high specificity and a positive likelihood ratio greater than 10, suggesting that a raised result meaningfully increases the probability of complicated disease within this selected operative population. WBC was less discriminative, although its NPV remained relatively high.

The observed association is biologically plausible. Advanced appendiceal inflammation may increase portal exposure to bacteria and endotoxins, while systemic cytokine responses can reduce hepatocellular bilirubin transport and produce transient cholestasis [10,11]. Bilirubin therefore may reflect the severity of the inflammatory process rather than appendicitis itself. This interpretation is consistent with the weaker performance observed when uncomplicated appendicitis was compared with a macroscopically normal appendix.

Our findings are stronger than several previous estimates. Sand et al. reported higher specificity for bilirubin than leukocytosis in perforated appendicitis, although bilirubin was less sensitive [12]. A diagnostic meta-analysis by Giordano et al. found more modest pooled sensitivity but retained evidence that hyperbilirubinemia increased the likelihood of perforation [13]. Earlier studies of leukocyte count and neutrophil percentage also support the role of inflammatory markers as adjuncts rather than standalone tests in suspected appendicitis [7,8]. Differences across studies may result from case selection, definitions of complicated disease, bilirubin thresholds, timing of blood sampling, prevalence of perforation or gangrene, and whether histopathology was used as the reference standard.

The high performance in the present study should therefore be interpreted cautiously. The optimal threshold was selected and evaluated in the same dataset, which can overestimate accuracy. In addition, all participants underwent surgery, so the results may not apply to patients discharged after observation or treated non-operatively. Bilirubin was not evaluated within a multivariable model containing symptom duration, C-reactive protein (CRP), clinical score, or imaging features; consequently, independent incremental value beyond routine assessment has not been established.

From a clinical perspective, bilirubin should not be used as a standalone indication for appendicectomy. A raised value may support concern for gangrene, perforation, abscess, or peritonitis when interpreted with the history, examination, inflammatory markers, and imaging. Conversely, a normal bilirubin cannot safely exclude complicated disease. Future studies should validate the threshold prospectively, use standardized operative and histopathological criteria, and assess whether adding bilirubin to a clinical-imaging model improves calibration, decision-making, and patient outcomes.

Limitations

The study was conducted at one tertiary center and included only 29 complicated cases, resulting in relatively wide CIs. The cohort was restricted to operated patients, introducing selection and spectrum bias. Operative macroscopic assessment was used as the reference standard because histopathology was not routinely available, and this may have led to outcome misclassification. The cut-offs were derived and tested in the same cohort without internal optimism correction or external validation. CRP, symptom duration, and standardized cross-sectional imaging were unavailable for adjusted analysis. The negative appendicectomy rate was high, reflecting the local diagnostic pathway and limiting generalizability to centers using routine CT. Finally, occult causes of bilirubin elevation may have remained despite the exclusion criteria.

## Conclusions

Admission total serum bilirubin demonstrated good discriminatory performance for complicated appendicitis in this single-center operative cohort and outperformed WBC alone. It may be useful as an adjunctive risk marker, but it should be interpreted together with clinical and imaging findings. Independent prospective validation is required before the threshold is incorporated into treatment algorithms.
